# Overdispersion in COVID-19 increases the effectiveness of limiting nonrepetitive contacts for transmission control

**DOI:** 10.1073/pnas.2016623118

**Published:** 2021-03-19

**Authors:** Kim Sneppen, Bjarke Frost Nielsen, Robert J. Taylor, Lone Simonsen

**Affiliations:** ^a^Niels Bohr Institute, University of Copenhagen, 2100 København Ø, Denmark;; ^b^Department of Science and Environment, Roskilde University, 4000 Roskilde, Denmark

**Keywords:** pandemic, overdispersion, mitigation strategies, superspreading, social networks

## Abstract

Increasing evidence indicates that superspreading plays a dominant role in COVID-19 transmission. Recent estimates suggest that the dispersion parameter *k* for severe acute respiratory syndrome coronavirus 2 (SARS-CoV-2) is on the order of 0.1, which corresponds to about 10% of cases being the source of 80% of infections. To investigate how overdispersion might affect the outcome of various mitigation strategies, we developed an agent-based model with a social network that allows transmission through contact in three sectors: “close” (a small, unchanging group of mutual contacts as might be found in a household), “regular” (a larger, unchanging group as might be found in a workplace or school), and “random” (drawn from the entire model population and not repeated regularly). We assigned individual infectivity from a gamma distribution with dispersion parameter *k*. We found that when *k* was low (i.e., greater heterogeneity, more superspreading events), reducing random sector contacts had a far greater impact on the epidemic trajectory than did reducing regular contacts; when *k* was high (i.e., less heterogeneity, no superspreading events), that difference disappeared. These results suggest that overdispersion of COVID-19 transmission gives the virus an Achilles’ heel: Reducing contacts between people who do not regularly meet would substantially reduce the pandemic, while reducing repeated contacts in defined social groups would be less effective.

## Significance

Evidence indicates that superspreading plays a dominant role in COVID-19 transmission, so that a small fraction of infected people causes a large proportion of new COVID-19 cases. We developed an agent-based model that simulates a superspreading disease moving through a society with networks of both repeated contacts and nonrepeated, random contacts. The results indicate that superspreading is the virus’ Achilles’ heel: Reducing random contacts—such as those that occur at sporting events, restaurants, bars, and the like—can control the outbreak at population scales.

Countries worldwide have responded to the COVID-19 pandemic by implementing unprecedented “lockdown” strategies: closing schools and workplaces; closing or strictly regulating restaurants, bars, theaters, and other venues; and banning large gatherings. Such measures moderately reduced disease transmission in the 1918 Spanish influenza epidemic (1); however, in the COVID-19 pandemic, lockdowns have been highly effective, albeit at great cost to society (2). Not enough is known about which of the mitigation measures used during lockdowns is most effective. Understanding the relative contributions of reducing different types of contacts in different settings is essential for the current situation as well as for pandemic preparedness.

The occurrence of “superspreading events,” in which a large number of people are infected in a short time (often in a single location), is a well-documented aspect of the COVID-19 pandemic (3), from a string of superspreading events at fitness centers in Seoul, South Korea (4) to a wedding reception at the Big Moose Inn in Millinocket, ME at which at which over half the guests were infected (5).

Heterogeneity in transmission is well known in several infectious diseases (6–9), including the recent coronavirus threats severe acute respiratory syndrome (SARS) (10) and Middle East respiratory syndrome (MERS) (11). In 2005, Lloyd-Smith et al. (6) surveyed the importance of superspreading events across infectious diseases and pioneered the use of the “dispersion parameter” *k* to describe how the number of infections generated by an individual is distributed around the mean, with lower values of *k* corresponding to a broader distribution.

Multiple studies have found that *k* for SARS-CoV-2 is on the order of 0.1, corresponding to ∼10% of infected people causing 80% of new infections (12–15) This also implies that the majority of infected individuals cause less than one secondary infection and thus, cannot sustain the epidemic on their own should the superspreading events somehow be prevented.

Consistent with this, the household attack rate is low, as shown by several studies. In China, figures of 15 and 12% have been reported (13, 16), while a nationwide study from Denmark gave a household attack rate of 17% (17); in the context of a superspreading event in a South Korean call center, the household attack rate was 16% (18). The low household attack rate implies that most infected people do not even infect their household contacts. The overdispersion seen in SARS-CoV-2 stands in contrast to pandemic influenza, which was found to have a dispersion parameter of about *k* = 1 (19), so that 45% of infected people cause 80% of new infections.

Measurements of the level of transmission heterogeneity in COVID-19 have been based on several different methodologies, each having its own strengths and weaknesses. Perhaps the most direct measurement is by contact tracing (13). This method allows for a straightforward assessment of overdispersion but may be affected by biases inherent in contact tracing data, such as close contacts being more readily found or large outbreaks being more carefully investigated. Other studies have relied on aggregate incidence data (12, 15, 20) and even phylodynamic methods (14). These disparate studies found similar levels of heterogeneity, increasing the robustness of the basic finding that overdispersion is high in COVID-19.

Given the importance of superspreading to COVID-19 transmission, modeling studies assessing the effect of different mitigation strategies would do well to take superspreading into account. Agent-based models, which set up a network of individual agents that interact according to defined rules, are well suited to exploring the impact of mitigation in the presence of superspreading. Like standard compartmental Susceptible, Exposed, Infected, Recovered (SEIR) models, they can reproduce the epidemic curves observed in a population in an unmitigated scenario. Unlike purely compartmental models, agent-based models can easily adjust individual infectivity and mimic repeated social interactions within defined groups, as might be found in households, schools, and workplaces. Agent-based models can also include different types of social interaction and phenomena such as a disease saturating some households or workplaces by infecting all susceptible agents.

**Fig. 1.**
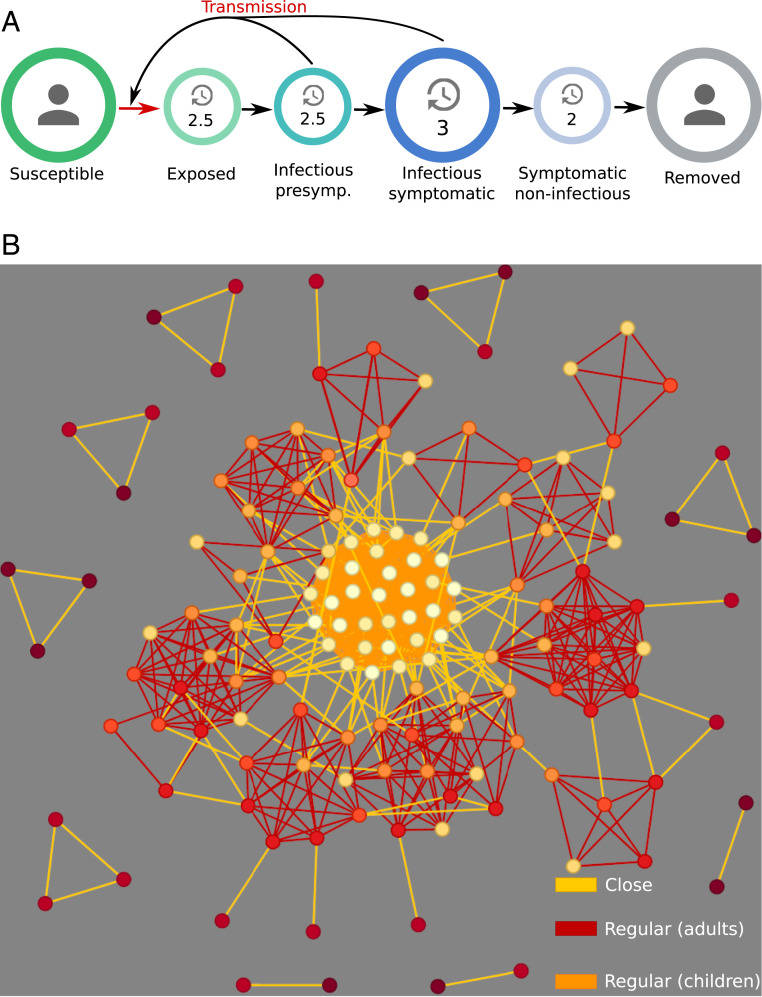
(*A*) Schematic representation progression of disease in our agent-based model. Individual agents become infectious 2.5 d before symptom onset on average. Agents enter the recovered state after an average of 3 d of symptoms, giving an average total infectious period of 5.5 d. (*B*) Schematic representation of the connectivity between 150 agents. Individuals are represented as nodes, with shading indicating age (light = young, dark = older). Edges represent social connections, with bright yellow denoting close contacts, orange denoting regular contacts between adults, and red edges denoting regular contacts involving children. Random contacts are not pictured. The network diagram was generated by running our simulation on a smaller population of just 150 individuals, with the same rules for connectivity as in the full-scale simulations.

We therefore developed an agent-based model with a social network structure to investigate how overdispersion might affect nonpharmaceutical mitigation efforts to control a superspreading disease such as COVID-19. In brief, we simulated epidemic trajectories in an agent-based model with a population of 1 million agents. Upon infection, agents transition from susceptible to exposed, infected, and recovered states (Fig. 1*A*); agents are on average infectious for 5.5 d. We allowed contacts of three types: close (within a small, unchanging group as might be found in a household or other close association), regular (within a larger, unchanging group as might be found in a workplace, school, extended family, or other social unit), and random (drawn randomly from the entire agent population and not repeated regularly) (Fig. 1*B*). We adjusted the contact rates to achieve a 1:1:1 ratio of contact time in the three sectors, consistent with survey data from Mossong et al. (21). Within the timescale set by the generation time of COVID-19, our close and regular networks can be considered constant. Contacts that occur less frequently belong to the random sector. To simulate superspreading, we assigned infectivity according to a gamma distribution with dispersion parameter *k* = 0.1 and adjusted the overall infectivity to produce an initial growth rate of 23% per day, as observed for COVID-19 in Europe and North America (22–24), which corresponded to a basic reproductive number of 2.5. In the unmitigated case, contacts were allowed in all three sectors; we then simulated two additional scenarios in which the regular and random contacts were restricted. These three scenarios were simulated under two conditions, with *k* set to infinity (no superspreading) and with *k* set to 0.1 (superspreading). The model is described in detail in *Methods*.

Our findings suggest that superspreading gives COVID-19 an Achilles’ heel: Limiting contacts in the part of the social environment where many random contacts are encountered—and where superspreading events are most likely to occur—slows transmission dramatically and far more effectively than limiting contacts in social groups where people meet repeatedly, such as in the home, work, or school.

## Results

**Fig. 2.**
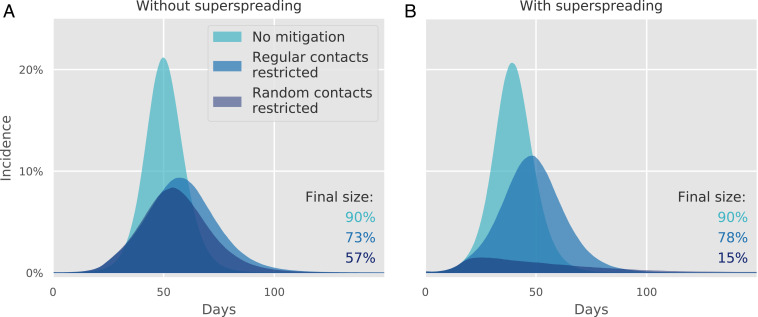
The impact of mitigation on modeled incidence. Simulation of epidemic trajectories with mitigation starting when 1% of the population has been infected. In each panel, we show three trajectories corresponding to the unmitigated epidemic, the case where we completely restrict all regular contacts, and the case where we restrict all random contacts. When superspreading is not present (i.e., *k* is infinite; *A*), the effect of eliminating regular and random contacts is similar; however, when superspreading is a factor in transmission (i.e., when *k* = 0.1; *B*), the effect of eliminating random contacts is dramatically enhanced. We did not consider mitigation by limiting close contacts as this would not be a credible mitigation strategy.

We found that the presence of superspreading profoundly improves the impact of reducing random contacts in mitigating the epidemic. Regardless of whether superspreading is present in the model, the overall percentage of the population infected in a no mitigation scenario is 90% (Fig. 2). Thus, superspreading has hardly any effect on the trajectory of an unmitigated epidemic. Furthermore, comparing Fig. 2, it is clear that a mitigation strategy based on restricting regular contacts performs similarly in both the superspreading (Fig. 2*B*) and nonsuperspreading (Fig. 2*A*) scenarios. However, when a mitigation strategy based exclusively on restricting random contacts is employed in the superspreading scenario, the effect is dramatically enhanced: The final epidemic size is just 15%, compared with 57% in the absence of superspreading.

We performed several sensitivity tests to investigate whether our findings were robust to changes in model parameters.

**Fig. 3.**
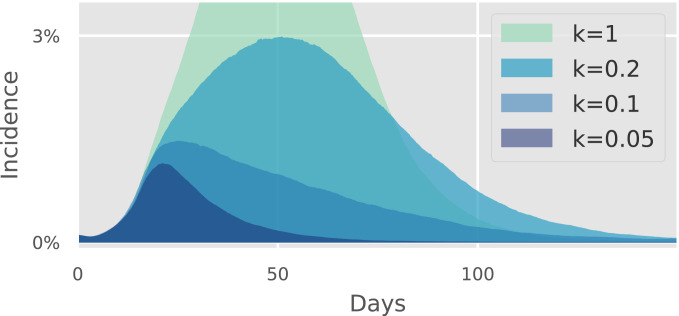
Sensitivity of model results to dispersion factor *k*. When an epidemic size of 1% is reached, a mitigation scheme consisting of restricting all random contacts is initiated. We explore the epidemic trajectories for different values of the overdispersion factor *k*. As *k* decreases (i.e., transmission heterogeneity increases), eliminating random contacts has a progressively greater effect.

We varied the dispersion parameter *k* in the interval [0.05, 1.0] and found that as it increased, the effect of preventing random contacts gradually diminished (Fig. 3). This shows that the efficacy of random sector-based mitigation increases monotonically with the degree of superspreading. On the other hand, even partial mitigation of the random sector still had a considerable effect when *k* = 0.1 (*SI Appendix*, Fig. S1).

By adjusting the mean infection rate, we varied the initial epidemic growth rate from 16 to 30% per day (*SI Appendix*, Fig. S2), an interval that covers the range of premitigation growth rates observed in Europe and North America (22–24). We found, as expected, that a faster-growing epidemic is more difficult to mitigate; however, the enhanced effect of random sector mitigation when superspreading is present remains.

To assess the sensitivity of our results to the partitioning of the three social sectors, we varied the ratio of contacts in each sector from the base case of 1:1:1 to 2:2:1 for close, regular, and random contacts (*SI Appendix*, Fig. S3*A*) and increased the size of the groups from which regular and close contacts were drawn, respectively (*SI Appendix*, Fig. S3 *B* and *C*). These variations had only a moderate negative effect on mitigation, reflecting that a mitigation strategy based on removing random contacts becomes relatively less effective if fewer random contacts are made in the premitigation scenario. In a related analysis, we analyzed the effect of introducing heterogeneity in the number of individuals with whom an agent interacts. We did this by letting half of the population spend only 1/6 of their contact time in the random sector while allowing the other half to spend 1/2 of their contact time interacting in the random sector. In this way, we maintained the overall activity in the random sector to be 1/3. The result was a moderate decrease in the degree of mitigation (*SI Appendix*, Fig. S4).

To determine the effect of increased heterogeneity in social activity, we exponentially distributed the overall contact time of individuals, so that some agents would make contact more frequently than others (*SI Appendix*, Fig. S5). This heterogeneity was found to decrease the epidemic size in general, similar to what Britton et al. (25) recently showed for COVID-19. Nonetheless, random sector-based mitigation remained by far the most effective.

Finally, we measured the distribution of the number of secondary infections arising in our simulations (*SI Appendix*, Fig. S6). This analysis is an important test of our model since it is crucial that the model reproduces the degree of transmission heterogeneity reported in the literature; the analysis also allows us to assess the degree of transmission heterogeneity introduced by the model’s social structure alone. When we set the dispersion parameter for infectivity to *k* = 0.1 (our base superspreading scenario), the coefficient of variation (*CV*) of the observed distribution of secondary cases is 3.1, consistent with an observed *k* value of ∼0.1 for a negative binomial distribution (6), indicating that the model has the desired level of transmission heterogeneity in our base superspreading scenario. When the distribution of infectiousness is taken to be homogeneous (i.e., the nonsuperspreading scenario [formally obtained at infinite *k* for infectivity]), the observed distribution of cases has a *CV* of 0.7, consistent with an observed *k* value of 3.3 for a corresponding negative binomial distribution. Thus, the social structure by itself contributes only very moderately to the transmission heterogeneity observed in our superspreading simulations.

Across the sensitivity analyses, our basic finding remains unchanged: In an epidemic driven by superspreading, restricting random nonrepeating contacts is far more effective than limiting the regular repeating contacts that occur in interconnected groups.

## Discussion

Policy makers worldwide face excruciating choices as they seek to ease restrictions as much as possible without causing a surge in COVID-19 cases that would overwhelm health care systems, especially by exceeding available intensive care unit beds needed to keep critically ill COVID-19 patients alive. These policy choices must take new information into account as the pandemic unfolds.

Evidence is now overwhelming that superspreading plays a key role in COVID-19 transmission (12–15). Yet, models used to predict effects of mitigation strategies often do not consider this phenomenon (26–28). In this study, we built an agent-based model with an underlying social structure to take on this task.

Our results indicate that reducing random contacts has an outsized effect in an epidemic characterized by superspreading; in the absence of superspreading, the same mitigation strategy is much less effective. This means that mitigation policies should focus on limiting contacts during activities that bring together large numbers of people who would otherwise not routinely come into contact, such as at sporting events, restaurants, bars, weddings, funerals, and religious services; repeated contacts that occur in smaller social groups are much less important. If such gatherings cannot be avoided, steps such as wearing face masks and moving events outdoors might also help. Our results also suggest that in complex settings such as workplaces and schools, which have characteristics of both our regular and random sectors, preventing congregation of large groups of people who would otherwise rarely meet is important.

Why does our model suggest that the presence of superspreaders favors these policy choices? When random contacts are prevented, regular contacts become the main source of infection. However, because the number of possible connections is limited in a regular social unit, a highly infectious individual soon runs out of susceptible contacts. When random contacts are allowed, however, there is no such limitation because as far as the superspreading agent is concerned, every contact is new. It follows that an epidemic driven by superspreading is fueled more by the diversity of contacts—the total number of different people encountered—and less by the duration of contacts—how long one spends with each. Thus, preventing random contacts in the model provides more benefit than preventing regular contacts.

It is worth noting that an equal ratio of contact time across sectors does not mean that the number of secondary infections is the same in each. Even when *k* is high so that superspreading is not present (Fig. 2*A*), about 40% of transmissions occur during random contacts because the saturation effect is small. When *k* is low and superspreading is present, this fraction increases to about 60%, the removal of which corresponds to a 2.5-fold reduction in the reproductive number of the disease—a reduction sufficient to mitigate the epidemic (Fig. 2*B*).

Our finding that the propagation of an overdispersed disease is more sensitive to the many random contacts (rather than the few but persistent regular contacts) is broadly applicable, regardless of the underlying biological mechanism. If, for example, one considers a disease where the high reproductive number of some individuals is the result of a prolonged infectious period, transmission would still be limited by the number of different persons an individual encounters. In our model, this number is set by the combined size of their close and regular contacts, when access to random contacts is restricted.

The most important limitation of our study is the model’s simplicity compared with the complex reality of human society. Our social structure does not precisely reproduce the complex and fluid interactions of human societies. However, our division of contacts approximates the range of possible interactions, from familiar to random. We relegated all nonrepeating contacts to the random sector, so that contacts with known persons occurred only through two fixed social networks, one small and one somewhat larger. In the real world of large families, workplace cafeterias, school playgrounds, and neighborhood restaurants, many interactions in the random sector would be with familiar but rarely seen people such as old friends and extended family; likewise, some contacts with random people would occur in places dominated by repeat contacts with familiar people. We simply separated those into two artificially distinct spheres.

The mechanism that underlies superspreading is not understood, but relevant factors include both the rate at which an infected person sheds the virus and the environment in which the virus is shed, including the density of people and their susceptibility. Behavior, including shouting or singing, can increase both the rate of viral shedding and the susceptibility to infection, and a gathering in a closed room with poor ventilation involves considerably higher risk than one outdoors (29, 30). Superspreading has been broadly categorized in three main categories: biological, behavioral/social, and opportunistic (31). However, these categories are not mutually exclusive, and superspreading is generally a question of means (high infectiousness) and opportunity (social and environmental context). In order for a superspreading event to occur, a highly infectious individual must have access to a large number of distinct contacts. In our model, the means is simulated by assigning a distribution of individual infectiousness from a gamma distribution. While we do not specifically model events, we do allow many contacts in the random sector, which allows some agents to cause large clusters of secondary infections.

Other recent studies modeling superspreading in COVID-19 have generally come to the conclusion that “cutting the tail” (i.e., targeted elimination of superspreaders) would be an effective means of mitigation (31–33). What is less clear is how to construct policies to accomplish that and how to identify the situations and modes of contact which are likely to lead to superspreading. By distinguishing between repeated and random contacts, our model points to a feasible population-wide mitigation strategy. This is not possible in well-mixed (32), branching process (31), or purely network-based models (33), which do not incorporate different types of social contacts.

The social network underlying our model is of the “small-world” variety (34), insofar as it is characterized by cliquishness and short typical distances between nodes. Thus, any given node in our model can typically be reached by moving through only a few close and regular units. Block et al. (27) recently used small-world networks to explore how mitigation strategies that alter typical nodal distance and cliquishness affect the epidemic trajectory. In the same vein, Leng et al. (35) studied the influence of social bubbles on mitigation efforts using an agent-based model with three levels of transmission: within households, between households in the same bubble, and lastly, community spread (akin to our random sector). However, none of these papers addressed the effect of superspreading on the mitigation strategies. Our results lend support to mitigations based on cutting links between cliques (27, 35) since the mixing of different close and regular groups occurs primarily through encounters in the random sector. Our work further shows that this kind of mitigation strategy is enhanced in a pandemic characterized by superspreading, as illustrated by Fig. 2*B* (compared with Fig. 2*A*).

Superspreading is a defining feature of the COVID-19 pandemic; a relatively small minority of the population causes the majority of infections, while most do not even infect people in their own household. As it is not possible to identify these superspreaders before transmission occurs, we here suggest an effective alternative strategy, namely that policies should aim to reduce contact diversity, rather than attempt to limit total contact time. This means that mitigation policies should focus on limiting activities that bring together many people who would otherwise not routinely come into contact.

## Methods

We developed an age-stratified, agent-based model with three sectors of social contact through which the disease can be transmitted. Each agent is assigned to one close and one regular unit, within which contacts are repeated over time, and participates in random contacts drawn from the entire population.

Agents are stratified by age in 10-y intervals and assigned age-dependent social activity levels *a*_*i*_, which are adjusted such that the observed contact rates in an unmitigated scenario fit the age-dependent activity given in Table 1 (21). Close units have some properties of households: an average of 2.3 members, adults are in the same or adjacent age bands, and children are taken to be 20 to 40 y younger than adults in the same unit. The *CV* of the generated close contact network sizes is 0.59. This may be compared with The European Union Statistics on Income and Living Conditions Survey, which reports an average household size of 2.3 with a *CV* of 0.57 (36). Regular units have properties of workplaces and schools: Agents 20 to 70 y of age are assigned to a Poisson-distributed cluster with an average of eight agents. Agents under 20 y old are assigned a regular unit of 18 members. Each of these units is also assigned two adults aged 20 to 70. Agents older than 70 y are not assigned to a regular unit. Random contacts are chosen from the entire population at random for each infection attempt to simulate brief contacts without temporal correlation.

**Table 1.** Distribution of simulated population by age group (40), with conditional probabilities for relative social contact time (21)

Age (y)	Percentage of population	Relative social time per person
0–9	10.9	1.21
10–19	11.9	1.70
20–29	13.3	1.45
30–39	11.7	1.45
40–49	13.6	1.38
50–59	13.6	1.31
60–69	11.7	1.06
70–79	8.9	0.81
80+	4.3	0.81

The progression of the disease is modeled in an SEIR framework, with agents passing through each stage at a rate determined by the average durations given in Fig. 1. The exposed state is subdivided into four stages, each of 1.25 d in length, with a constant probability rate for transitioning from one stage to the next. The first two of these stages comprise the gamma-distributed preinfectious state (average total duration: 2.5 d, SD: 1.8 d). The next two stages comprise the presymptomatic infectious state (average total duration: 2.5 d, SD: 1.8 d). This is followed by the infected state, in which agents are infectious and symptoms may be displayed [average total duration: 3 d (37, 38), SD: 3 d]. Agents then pass into the recovered state where they are no longer infectious. Simulations are run in a population of 1 million, randomly seeded with 100 infected agents. Agents are assigned a gamma-distributed infectivity *β·s*_*i*_, where *s*_*i*_ is drawn from a gamma distribution *P*(*s*), proportional to *s*^*k*−1^ exp(−*k s*) with continuous *s* > 0 [Lloyd-Smith et al. (6)]. Here, *k* is the dispersion parameter, which determines the *CV* of the distribution according to *CV* = $1/\sqrt{k}$. The rate constant *β* is calibrated to reproduce the observed initial exponential growth rate of 23% per day of an unmitigated COVID-19 epidemic (22–24).

In each time step of size Δ*t* (of 30-min duration), each infected agent has an age-dependent probability for making contact to another agent; for each such contact, a contact partner is drawn from one of the three social sectors. The rate at which each of these sectors is chosen is based on a population-based survey of mixing patterns in eight European countries by Mossong et al. (21). That study found that the “home” sector made up 19 to 50% of all contacts, while the “work/school” sector accounted for 23 to 37%, and the remaining sectors amounted to 27 to 44%. For our model, we approximated this stratification by letting one-third of all contacts fall into each of the three sectors, for our base case. In *SI Appendix*, Fig. S2, we investigate the effect of varying these sector-specific social contact frequencies. Potential targets for infection are selected proportional to the age-dependent social activity listed in Table 1.

At each contact, the disease is transmitted with probability *P*_*t*_ = *β s*_*i*_ *Δt*. The time step length is chosen small enough to ensure that the probability of infection in any given time step is always less than one, even for the most infectious individuals. We simulate mitigation strategies by not permitting infection in a chosen fraction of contacts in one or more of the contact sectors. Mitigation is initiated when the infected population reaches 1% of the total. When mitigation by reduction of random contacts is performed, social networks are kept fixed, and the same numbers of contacts are removed in superspreading and nonsuperspreading scenarios to facilitate direct comparison.

To analyze the impact of heterogeneous social activity, we assigned each agent a separate activity parameter *a*_*i*_ selected from an exponential distribution (*SI Appendix*, Fig. S5). At each contact attempt from agent *i* to agent *j*, if *a*_*i*_ < *a*_*j*_ then the contact proceeds as usual; however, if *a*_*i*_ > *a*_*j*_, then the contact proceeds with a probability *a*_*j*_/*a*_*i*_. This procedure yields an exponential distribution of observed social activity, with more active agents being removed from the susceptible pool earlier in the epidemic.

## Supplementary Material

Supplementary File

## Data Availability

Model code data have been deposited in GitHub (https://github.com/NBIBioComplexity/SuperCoV) (39).
