# Supplementary material for: Overdispersion in COVID-19 increases the effectiveness of limiting nonrepetitive contacts for transmission control
Source: Proc Natl Acad Sci U S A. 2021 Mar 19;118(14):e2016623118. doi: 10.1073/pnas.2016623118 (PMC8040586; doi:10.1073/pnas.2016623118)
Supplement: Supplementary File [file pnas.2016623118.sapp.pdf]

Supplementary Figures

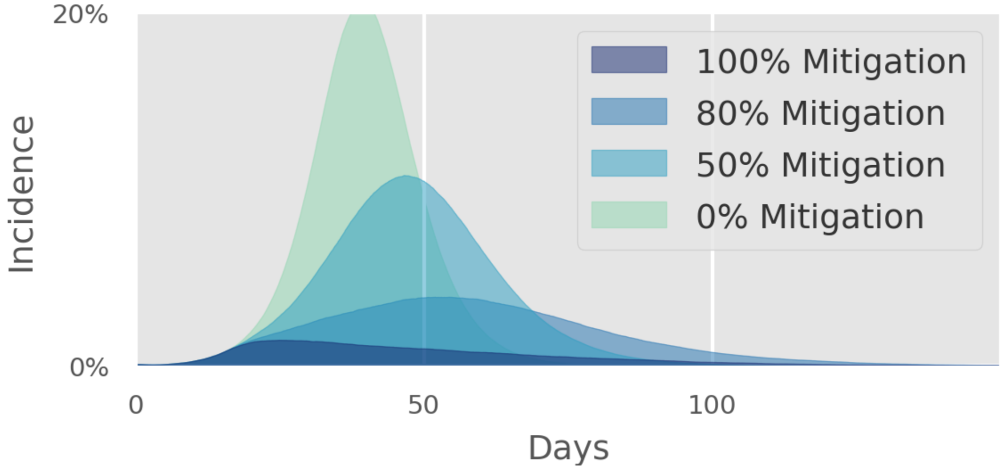

Figure S1. Sensitivity of results to partial elimination of random contacts. In the mitigation scenario shown in the main text (Figure 2), all random contacts are eliminated when mitigation commences. Here we show how the efficacy of mitigation depends on the fraction of random contacts which are eliminated.

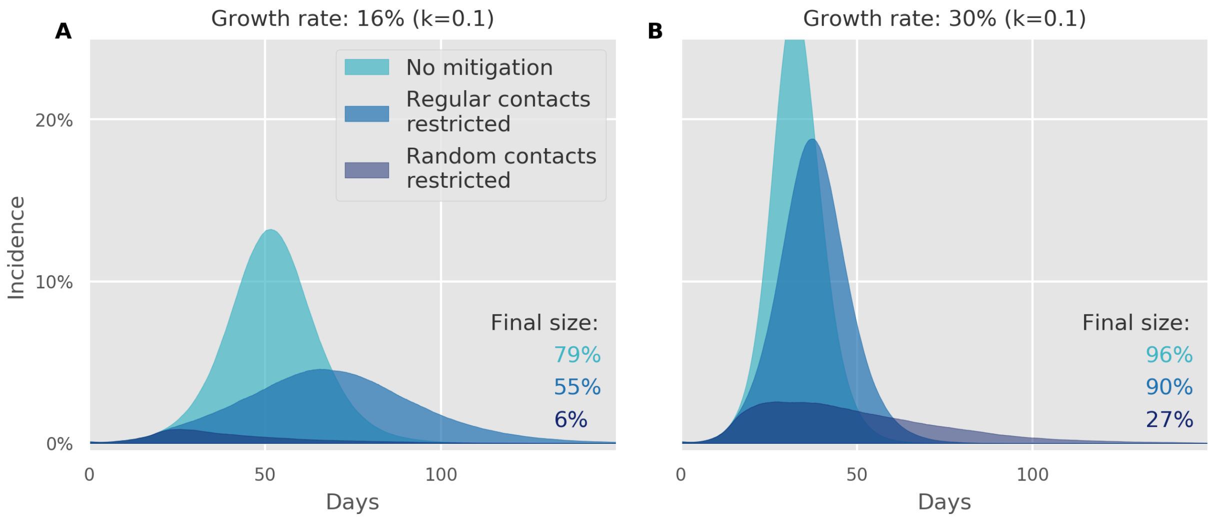

Figure S2. Sensitivity of results to initial growth rate. In the main text, we assumed an initial daily growth rate of 23%. This was based on estimates for Italy, but variations between countries and regions have been reported. Here we show the effect of mitigation strategies based on restricting *regular* or *random* contacts when the initial growth rate of the epidemic is 16% **(A),** and 30% **(B)**. In both cases the dispersion parameter is *k=0.1* and we start mitigation when an epidemic size of 1% has been reached.

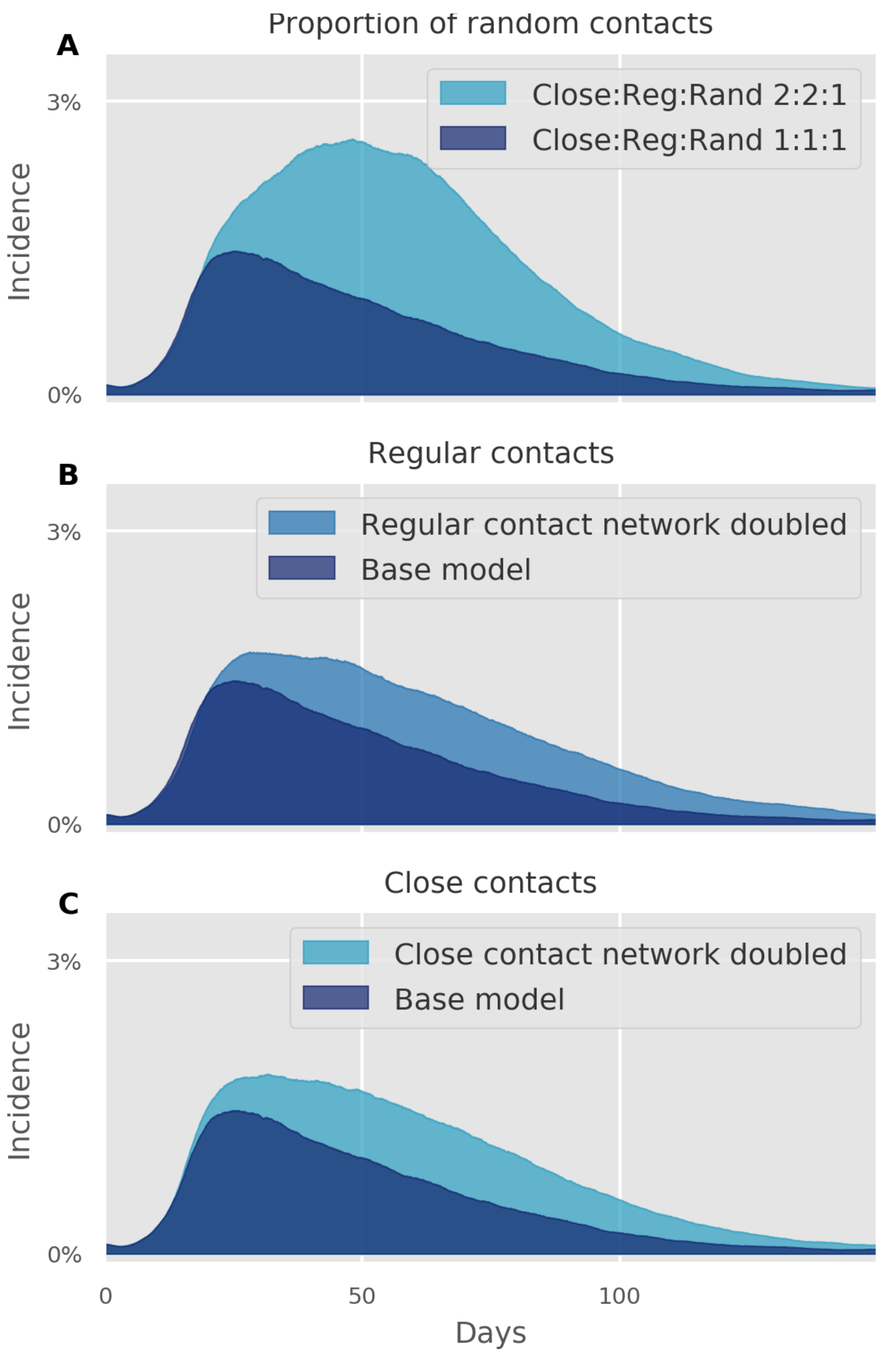

Figure S3. Sensitivity of results to the number of *random* contacts (A), size of *regular* contact circle (B) and size of *close* contact circle (C). In all cases we use overdispersion factor *k=0.1* and start mitigation when 1% of the population has been infected. Panel **A)** compares our base case, where 33% of contact time is spent in the random sector, to a case where only 20% contact time is in the random sector (with all percentages referring to pre-mitigation levels). Panel **B)** shows the effect of doubling the size of the *regular* circle of contacts, for alle age groups. Panel **C)** shows the effect of doubling the number of close contacts, from the base level of 2.3 persons.

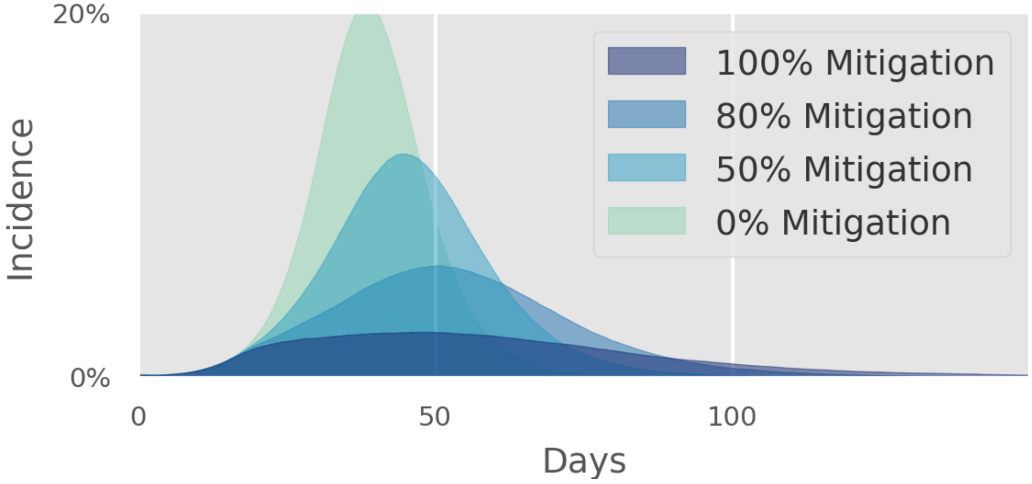

Figure S4. Sensitivity of results to introducing heterogeneity in the number of *random* contacts. In the base model, all individuals spend one third of their social time in the *random* sector. In this figure, we have introduced heterogeneity in the amount of contact time spent in the random sector, and thus the total number of distinct contacts encountered. Here, half of the population now spends half of their time in the random sector, while the other half spends just one sixth of their time there. Comparing with Figure S1, this shows that mitigation by restriction of random contacts becomes somewhat less effective with increased heterogeneity in the number of random contacts, but that the effect is still substantial.

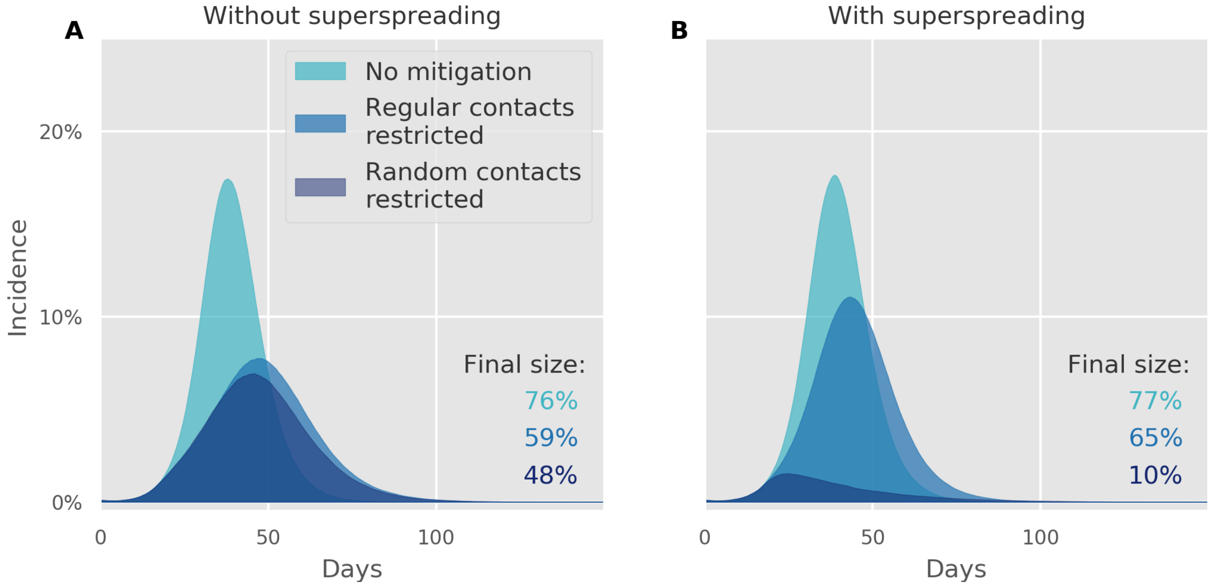

Figure S5. Sensitivity of results to heterogenous social activity. Here, the social activity levels of individuals are taken to be exponentially distributed, while still calibrating the initial growth rate of the epidemic to 23% per day. The overall attack rate is reduced because the least social people dominate the later stage of the epidemic. The effect of restricting random contacts is still moderate when there is no superspreading **(A)**, but strong when we introduce an overdispersion of infectivity with $k = 0.1$ **(B)**.

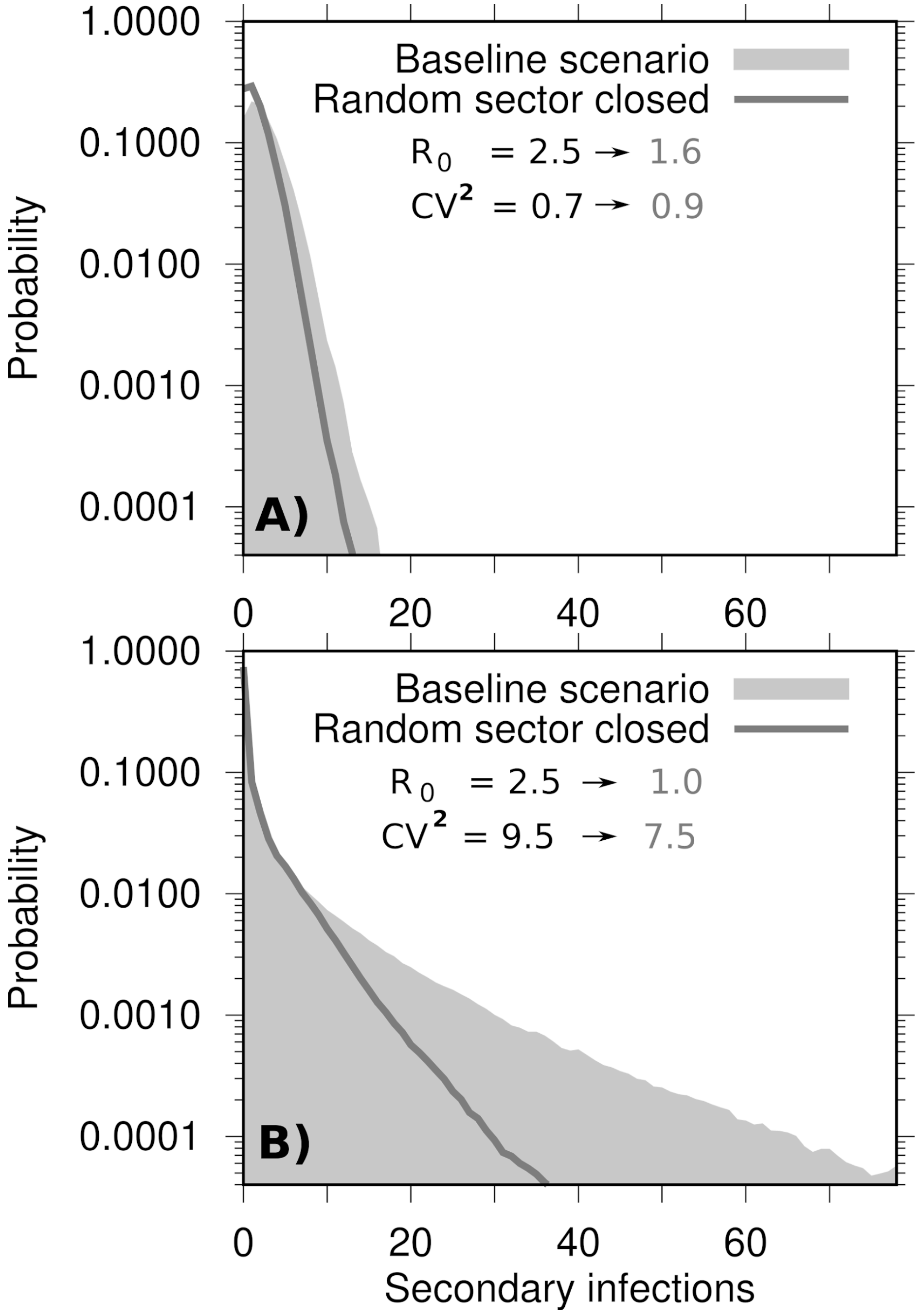

Figure S6. The distribution secondary infections arising in our simulations from an infected agent when the dispersion parameter *k* of the infectiousness distribution is set to **A)** infinity, corresponding to an evenly distributed underlying infectiousness, and **B)** *k = 0.1*, our base superspreading case. In each panel, the baseline scenario without mitigation (light grey shaded area) and the scenario with mitigation by restriction of random contacts (dark grey curve) are shown.
